# Nutritional Composition of Infant Cereal Prototypes Can Precisely Predict Their Glycemic Index

**DOI:** 10.3390/nu14183702

**Published:** 2022-09-07

**Authors:** Cathriona Monnard, Andreas Rytz, Carmen Mirela Tudorica, Gina L. Fiore, Tram Anh Line Do, Kalpana Bhaskaran, Katherine Macé, Yasaman Shahkhalili

**Affiliations:** 1 Nestlé Institute of Health Sciences, Société des Produits Nestlé S.A., 1015 Lausanne, Switzerland; 2 Nestlé Research Center, 1000 Lausanne, Switzerland; 3 Nestlé Product Technology Center, Société des Produits Nestlé S.A., Route de Chavornay 3, 1350 Orbe, Switzerland; 4 Centre for Applied Nutrition Services, Glycemic Index Research Unit, School of Applied Science, Temasek Polytechnic, Singapore 529757, Singapore

**Keywords:** glycemic index, glycemic load, infant cereals, macronutrient composition, model

## Abstract

Designing cereal-based products with appropriate metabolic responses is of high interest to the food industry in view of the potential health impact of the product. The objective of this study was to test whether a model that used the nutrient composition of breakfast cereals to predict their glycemic index (GI) and glycemic load (GL) could also accurately predict the GI and GL for complete (containing protein, reconstituted in water) infant cereal prototypes. Four independent studies measured the postprandial glucose response of 20 complete infant cereal prototypes (51–76 g/100 g glycemic carbohydrates) in healthy adults. The predictions were strongly correlated with the measured values for both the GI (r = 0.93, *p*-value < 0.01) and GL (r = 0.98, *p*-value < 0.01). The in vivo incremental area under the curve (iAUC) for glucose showed a strong linear relationship with the predicted GL (r = 0.99, *p* < 0.01). In summary, the model previously developed to predict the GI and GL of breakfast cereals was both accurate and precise for infant cereals and could be considered a simple tool to support nutritionally responsible product development.

## 1. Introduction

Cereal grains are a major component of the adult diet since they contribute important nutrients [1]. Likewise, in infants, grains, including fortified infant cereals, can play an important role in meeting nutrient needs during the complementary feeding period [2]. At around six months of age, the introduction of complementary feeding is recommended, as exclusive breastfeeding is no longer sufficient to meet all the nutrient needs of an infant [3]. Infant cereals are among the first complementary foods introduced in many countries and provide a source of energy, carbohydrates, proteins, vitamins and minerals [4].

Cereals are an important source of glycemic carbohydrates, which are the carbohydrates in foods that are digested, absorbed and metabolized [5], and thus, can impact postprandial glucose responses (PPGRs). In adults, data from observational studies suggest that high postprandial glycemia is implicated in the development of obesity and chronic metabolic diseases, such as type 2 diabetes and cardiovascular disease [6]. Although data among younger age groups are limited, the PPGR to infant cereal may potentially have a long-term health impact on infants. Therefore, knowledge of how to modulate the PPGR to infant cereal would be useful.

The glycemic index (GI) and glycemic load (GL) of foods are impacted by carbohydrate type and quantity [7], as well as other macronutrients, such as protein [8], fat [9] and soluble viscous fiber [10]. It was shown that the GI and GL of breakfast cereals can be precisely predicted from their macronutrient composition [11]. The developed model quantifies both the impact of glycemic carbohydrates and the GI-lowering effect of other macronutrients. This model was designed to guide the food industry in the development of breakfast cereals and potentially other foods and beverages with improved PPGR while considering different uses and modes of preparation.

The aim of this work was to use data gathered in four studies performed between 2016 and 2019 during the development of complete infant cereals to test whether this model was also predictive for complete infant cereals prepared with water that considerably differ from breakfast cereals in many aspects. Regarding infant cereals, first, in terms of the nutrient composition, there are much higher levels of protein; second, there are changes in viscosity, which are known to impact the PPGR [12]; and third, it increases the serving size from a typical serving of breakfast cereal of 30 g to a serving of 50 g of infant cereal reconstituted in 250 mL water. A model to predict the GI of infant cereal prototypes would help to accelerate innovation through screening the metabolic impact of infant cereals at an early stage, thus reducing the number of prototypes to eventually be tested clinically.

## 2. Materials and Methods

**Figure 1:**
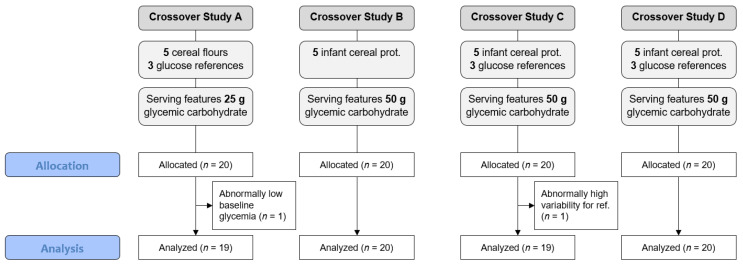
Flow chart showing the features of the four studies performed between 2016 and 2019. All samples were reconstituted in 250 mL water.

This paper presents the results of four independent in vivo studies that measured the PPGR of a total of 20 infant cereal prototypes. A flow chart providing information about each study can be found in Figure 1. Sixteen samples were aligned with the Codex Standards (i.e., to be prepared with water and contain adequate added protein) [13]. These samples are hereafter referred to as complete finished infant cereal prototypes (crossover study B–D). Additionally, four precooked, dried flour samples were included to understand the impact of the predominant ingredient of infant cereals on the GI and GL (crossover study A). To compare these 20 samples over all the product development steps, the PPGR was measured and expressed using the GI and GL [7]. The GI and GL are complementary. The GI characterizes the glycemic carbohydrates in a product, while the GL quantifies the postprandial impact on glycemia after the ingestion of a standard serving of a product. For example, a prototype has a GI of 58 if each gram of glycemic carbohydrate present in this prototype raised the blood glucose level similar to 0.58 g pure glucose. While a serving of the aforementioned prototype featuring 100 g of glycemic carbohydrates (GI of 58) will have a GL of 58, a smaller serving size of the same prototype with 50 g of glycemic carbohydrate reduces the GL to 29, which is comparable to a PPGR induced by 29 g of pure glucose.

### 2.1. Infant Cereal Prototypes

Twenty samples of either precooked, dried cereal flours (5 samples in study A) or finished complete infant cereal prototypes (15 samples in studies B, C and D) were characterized for their nutrient composition using standard analytical methods [14]. The model prototypes were predominantly wheat-based with various additions of other grains, sugars and dairy components. These model prototypes featured 51 to 76 g/100 g glycemic carbohydrates, 10 to 27 g/100 g proteins and 1 to 15 g/100 g fat (Table 1). The proportion of rapidly digestible starch was not assessed, but due to the processing conditions inherent to the manufacturing of infant cereals, the starch could be considered fully gelatinized, and therefore, fully digestible for all model prototypes.

### 2.2. In Vivo Trials

Four independent studies were conducted over different steps of product development between 2016 and 2019 at the accredited Glycemic Index Research Unit at Temasek Polytechnic, Singapore. The studies were approved by the Parkway Independent Ethical Committee (PIEC/2015/015 and PIEC 2017/009) in Singapore and were performed according to international standards [15]. The ISO standard specifies a method for the determination of the glycaemic index (GI) of carbohydrates in foods. It defines the GI, outlines qualifying factors and specifies requirements for its application. It also recommends criteria for the classification of foods into low, medium and high GI [15]. For these studies, the tests were performed on adults and not on infants for two main reasons: first, the standard methods of measuring the PPGR involve repeated blood sampling, which is not easily performed and ethically justified for infants or young children; second, at the time the studies were conducted, no minimally invasive continuous glucose monitoring device was validated for use on infants.

Each study consisted of 5 test samples and 3 glucose reference samples (except for study B, which did not feature the reference). Within each study, under fasting (10–14 h) conditions, 20 healthy subjects consumed all products in a crossover design, with one product per day and at least two days of washout between the test days. Two days was considered appropriate, as previous studies showed that even glycemic response testing on consecutive days did not seem to influence the variability of glycemic response tests compared with longer intervals and it did not cause any data drift under conditions of an earlier diet [16].

Two fasting, baseline (t = −5 min and t = 0 min) capillary blood samples were obtained. Following the second fasting blood sample, the product was consumed and the glycemic response was measured for 120 min postprandially, with blood being sampled at 15, 30, 45, 60, 90 and 120 min.

The subjects allocated to these studies were healthy males aged 21 to 55 years (mean = 28.4, SD = 9.1), with BMIs ranging between 18.5 and 26.1 kg/m^2^ (mean = 22.3, SD = 1.9) and average fasting glucose ranging between 3.6 and 4.7 mmol/L (mean = 4.2, SD = 0.25).

Since the four studies were designed at different steps of product development, the protocols were adapted to specific project needs, leading to different serving sizes or the addition of reference samples. These specificities are highlighted in the study flow chart in Figure 1, which also features the sample size of the allocated and analyzed subjects.

As can be seen from the study flow chart, the serving sizes of the infant cereal prototypes were adapted to adults and were thus greater than the typical serving size for infants. For study A, the portion size was lower than for the other studies to ensure the acceptability and tolerance of the flour samples.

The primary endpoints were the GI for the studies featuring the glucose reference (A, C and D) and the 2 h incremental area under the postprandial glycemia curve (2h-iAUC) for study B. The GI and GL were determined using commonly accepted equations [17]:

(1) $$\mathrm{GI}=\frac{2h-\mathrm{iAUC}\;\mathrm{of}\;\mathrm{test}\;\mathrm{sample}}{2h-\mathrm{iAUC}\;\mathrm{of}\;\mathrm{reference}\;\mathrm{sample}}\;\times \;100$$

(2) $$\mathrm{GL}=\frac{\mathrm{GI}\;\times \;\mathrm{amount}\;\mathrm{of}\;\mathrm{glycemic}\;\mathrm{carbohydrates}\;\mathrm{per}\;\mathrm{serving}\;\left[g\right]}{100}$$

In vivo measures of the 2h-iAUC, GI and GL are tabulated using the mean and standard error (SE) over all analyzed subjects in Table 2.

### 2.3. Model Predicting GI and GL from Nutrient Composition

The model previously developed to predict the GI of breakfast cereal as a function of its nutrient composition quantifies both the impact of glycemic carbohydrates and the GI-lowering effect of other macronutrients using a model that can be written as follows:

(3) $$\mathrm{GI}=\frac{\sum_{i=1}^{m}x_{i}GI_{i}}{\sum_{i=1}^{m}x_{i}+\sum_{j=1}^{n}x_{j}b_{j}}$$

In this equation, *x_i_* is the relative amount (g/100 g) of the *i*th glycemic carbohydrate (*i* = 1, …, m) and GI_i_ is its GI; *x_j_* is the relative amount (g/100 g) of the *j*th other macronutrient (*i* = 1, …, *n*) and *b_j_* is its GI-modulating power. The individual GI and GI-modulating power of the different nutrients are tabulated for all relevant nutrients [11]. For starch, the initial model introduced a correcting factor related to the rapidly digestible fraction; this correction was not necessary in this case since all starch could be considered fully digestible, leading to a GI of 100, equivalent to glucose.

As an example, using Equation (3), the GI of sample A1, which featured 2.1 g sucrose, 49.0 g starch, 9.4 g soluble fiber, 6.1 g insoluble fiber, 0.6 g fat, 27.0 g protein, 3.3 g ash and 2.5 g moisture, was predicted as follows:

eGI = (2.1 × 62 + 49.0 × 100)/[(2.1 + 49.0) + (9.4 × 0.3 + 6.1 × 0.1 + 0.6 × 0.6 + 27.0 × 0.6 + 3.3 × 0.1 + 2.5 × 0)] = 70

The predicted eGI of sample A1 was 70 as a result of featuring fully gelatinized starch (GI = 100) and sucrose (GI = 62); additionally, it took into account the GI-modulating powers that were different for soluble fibers (0.3), insoluble fibers (0.1), fat (0.6), protein (0.6), ash (0.1) and water (0). In this example, the calculation was made directly using the powder composition. The reconstituted product would lead to the same estimate since the GI-modulating power of water is considered null in the model.

The estimated GL (eGL) for a serving of 50 g was derived from the estimated eGI using the standard GL (Equation (2)). The amount of available carbohydrates in this sample was 51.1 g/100 g (with 2.1 g sucrose and 49.0 g starch), and therefore, 25.55 g for a serving of 50 g. Consequently, the predicted eGL for a serving of 59 g of sample A1 was as follows:

eGL = (70 × 25.55)/100 = 17.8 g glucose equivalent

Both the eGI and eGL were predicted independently of the in vivo data and could, therefore, be estimated for the model prototypes of study B for which no in vivo estimates of the GI and GL were available due to the absence of a reference.

### 2.4. Statistical Analyses

In vivo measures of the 2h-iAUC, GI and GL of individual prototypes are summarized using the mean and standard error (SE) to quantify the precision of the estimates (Table 2). Model predictions of the eGI and eGL are tabulated as point estimators. For studies where the in vivo estimates of the GI and GL were available (A, C and D), Bland–Altman plots were used to relate the in vivo estimates and model predictions [18]. These plots quantified the accuracy and precision of the model using, respectively, the mean and the standard deviation (SD) of the differences between predicted and in vivo mean estimates across all prototypes.

For study B, the eGL was compared with the incremental area under the curve (2h-iAUC) of the PPGR curves using a bivariate chart that represented means as dots and the standard errors (SEs) as error bars, as well as the Pearson correlation to quantify the strength of the linear relationship.

## 3. Results

### 3.1. Measured GI vs. Predicted GI

The in vivo GI of the 15 samples tested in studies A, C and D ranged between 62 and 91, with a mean of 72 and a standard deviation (SD) of 8.3. The three samples with a GI higher than 75 were precooked, dried flour samples. The predicted eGI of the same samples ranged between 62 and 89, with a mean of 72 and an SD of 9.1 (Table 2). The predicted eGI correlated strongly with the in vivo GI (r = 0.93, *p*-value < 0.01), and the model was not regressed to the mean, as shown by the fact that the range was the same for both the in vivo and predicted GI values.

**Figure 2:**
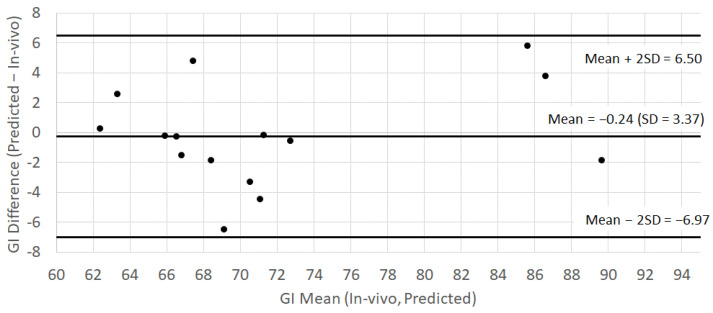
Bland–Altman plot comparing the in vivo GI estimates and eGI predicted from the nutritional composition of 15 samples, each of them being represented by a black dot (studies A, C and D).

The standard error (SE) of the in vivo GI estimates ranged between 3.4 and 5.4, with an average SE_pooled_ of 4.58. This standard error was larger than the standard deviation of the difference between the in vivo estimate and the model predictions, that is, SD = 3.37. The Bland–Altman plot for the GI (Figure 2) further revealed that the model prediction was accurate (i.e., the bias of −0.24 was negligible compared with SD = 3.37) and the precision of the prediction was independent of the magnitude of the GI. These results showed that the eGI predictions of the tested samples were both accurate and as precise as the in vivo predictions themselves.

### 3.2. Measured GL vs. Predicted GL

The in vivo GL of the 15 model prototypes ranged between 16.6 and 34.0 g of glucose equivalent with a mean of 22.6 g and an SD of 5.4 g. The predicted GL of the same model prototypes ranged between 17.8 g and 33.4 g with a mean of 22.6 g and an SD of 5.8 g (Table 2). The predicted eGL correlated strongly with the in vivo GL (r = 0.98, *p*-value < 0.01), and the model was not regressed to the mean, as shown by the fact that the range was the same for both the in vivo and predicted GL values.

**Figure 3:**
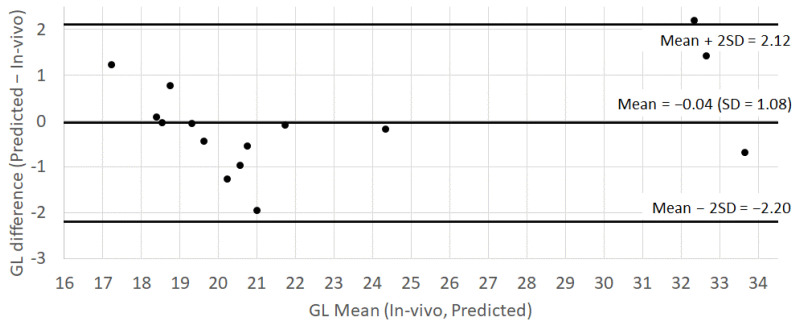
Bland–Altman plot comparing the in vivo GL estimates and the eGL predicted from the nutritional composition of 15 samples, each of them being represented by a black dot (studies A, C and D).

The standard error (SE) of the in vivo GL estimates ranged between 1.0 and 2.0, with an average SE_pooled_ of 1.43. This standard error was larger than the standard deviation of the difference between the in vivo estimate and the model predictions, that is, SD = 1.08. The Bland–Altman plot for the GL (Figure 3) further revealed that the model prediction was accurate (i.e., the bias of −0.04 was negligible compared with SD = 1.08) and the precision of the prediction was independent of the magnitude of the GL. These results showed that the eGL predictions of the tested samples were both accurate and as precise as the in vivo predictions themselves.

### 3.3. Measured PPGR vs. Predicted GL

**Figure 4:**
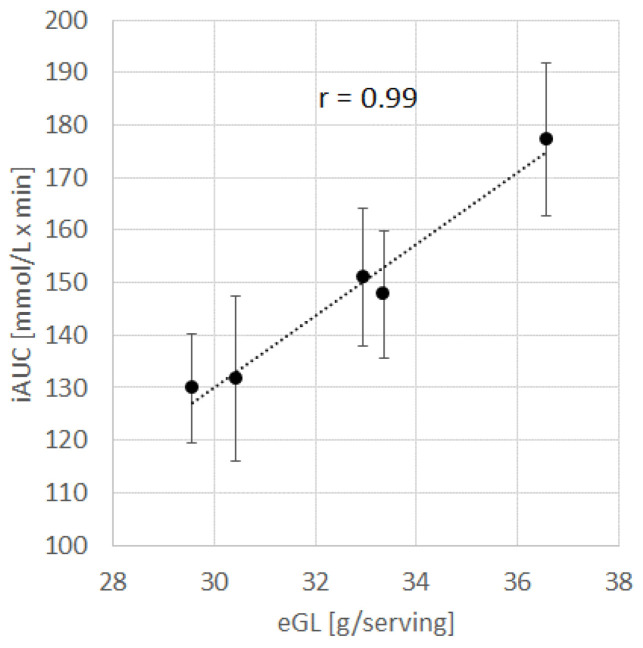
Relationship between the model-predicted eGL and the measured in vivo 120 min incremental area under the curve (iAUC) for the 5 infant cereal prototypes of study B, each of them being represented by a black dot (values are mean ± SE).

The five infant cereal prototypes of study B were not part of the previous results since neither the GI nor the GL could be estimated in vivo due to the absence of reference glucose during testing. Since the available carbohydrate content was similar between studies, it was possible to compare the in vivo incremental area under the curve (iAUC) with the predicted eGL (Figure 4). The linear relationship was very strong (r = 0.99, *p* < 0.01), and the in vivo variability was again larger than the imprecision of the prediction, as shown by the fact that the regression line crossed all error bars (mean ± SE).

## 4. Discussion

In the present study, we showed that a model that was previously used to predict the GI of breakfast cereals based on the nutritional composition could also predict the GI of complete infant cereal prototypes, which differed substantially in terms of the nutrient composition, processing and serving size to breakfast cereals.

### 4.1. Measured Glycemic Response to Complete Infant Cereal Prototypes

The 15 finished complete infant cereal prototypes (studies B, C and D) had GI values that ranged between 63 and 72, with a mean of 67. These values were close to the values reported in previous literature, which showed GIs of commercial infant porridges and gruel ranging from 67 to 75 [19] and were typical of such carbohydrate-based products. The GIs of the five precooked, dried flour samples used in study A were higher, ranging from 70 to 89, with a mean of 81, which was due to the fact that these flour samples consisted predominantly of starch (49–71%). The GI values of infant cereal prototypes obtained in the current study were lower than studies previously reported in the literature for hot cereals (porridges based on flour or flakes from barley and oats) intended for adults [20,21], which were found to have high GIs (91–105). The GI of food is impacted by several factors, most importantly by the type of carbohydrate (i.e., starch vs. sucrose), the levels of protein and fat, and the fiber that are more present in wholegrain than refined flours [22]. Additionally, processing can affect the starch crystallinity, which can alter the amylase availability and the GI [23]. Lastly, in the case of infant cereal, the addition of fruits (e.g., apple, pear) was also shown to lower the GI of porridges compared with a bread reference [19]. This is related to the lower GI of the fructose (GI = 20) compared with starch (GI = 100 if fully gelatinized), as well as the low-molecular-weight carbohydrates in the fruit being less available than the corresponding free sugars and, potentially, to the presence of organic acids (e.g., lactic acid and acetic acid) in fruit, which have been reported, at least in cereal-based products, to reduce glycemia [22,24] by reducing the gastric emptying rate [25] or affecting the starch bioavailability [26]. The PPGR is not only affected by the GI but also by the portion size, which is reflected in the GL of the product.

In the current study, the differences that we observed in the GI between the precooked, dried flour samples and the finished infant cereal prototypes were related to the macronutrient composition: the finished model prototypes contained significantly more fat, which can modulate the GI, while the flour samples in study A contained almost no fat but significantly more starch, which augmented the GI response. Higher protein contents further lowered the GI.

### 4.2. Validity of GI Predictions

The present results showed that the model previously developed to predict the GI and GL of breakfast cereals was also valid for infant cereals and could be considered a simple tool to accelerate nutritionally responsible product development that also applies in the case of infant cereals. The strength of the model lies in the fact that it quantifies both the impact of glycemic carbohydrates and the impact of GI-lowering nutrients, such as protein, fiber and fat, independent of their origin (plant or animal). It is remarkable that the model works similarly well for infant cereals and breakfast cereals considering the differences in nutrient composition, viscosity and serving sizes. This demonstrated that the model properly integrates changes in viscosity that are directly induced by nutritional composition and that it, therefore, needs no physical assessment of viscosity to deliver accurate and precise predictions.

Additionally, in the case of infant cereal prototypes, the model could reliably be used for the prediction of GI and appeared to be independent of small differences in the serving size of model prototypes (75–86 g for finished model prototypes, studies B, C and D) and the quantity of available carbohydrate (25 g in study A or 50 g in studies B, C and D). The results showed the relevance of the eGI and eGL as indices that need to be monitored during product development.

### 4.3. Relationship between eGL and PPGR

The results further showed that the eGL can be used as a good predictor of the PPGR. This observation was supported by previous studies in adults, which showed that meal GL is associated with incremental serum glucose and total AUC up to 5 h postprandially [27,28]. This association between the GL and GR was also demonstrated via continuous glucose monitoring in adults [29] and children [30] and was shown for both single foods and mixed meals [31]. According to a recent consensus statement, when considering the macronutrient composition, the GL/1000 kJ (239 kcal) is the single best predictor of the PPGR of foods [7]. Moreover, in healthy individuals, stepwise increases in GL were shown to predict stepwise elevations in postprandial blood glucose [32]. Given the current invasive nature of PPGR measurement in humans, the ability to use eGL from our current model as a proxy for this parameter would provide product developers with a non-invasive way to rapidly estimate the potential impact of their product on the PPGR for screening purposes. It is important to bear in mind that while we know the potential health impact of the PPGR for adults, further research is required to understand the potential applicability of the GI/GL concept in relation to the PPGR in infants/young children.

### 4.4. Application of the Model for Future Product Development

The results showed that the total amount of glycemic carbohydrates was insufficient to predict the GI, as exemplified by model prototypes C2 (65.3 g/100 g glycemic carbohydrates induced GI = 67 and eGI = 66) and D1 (57.0 g/100 g glycemic carbohydrates induced GI = 73 and eGI = 69). However, the effect of carbohydrates was very important for the GI and GL, and glycemic carbohydrates impacted the GI differently, with fructose (GI = 20), isomaltulose (GI = 32) and lactose (GI = 46) being less glycemic than sucrose (GI = 62), which was less glycemic than glucose, maltose or any digestible glucose polymer, such as fully gelatinized starch (GI = 100). Consequently, the complete infant cereal prototypes with the highest GIs were not those with the highest amounts of sucrose, but those with the highest amounts of glucose, maltose or starch. Other macronutrients, such as fat, protein and viscous soluble fiber, reduced the GI and, therefore, need to be considered in addition to glycemic carbohydrates. For example, a previous study showed that adding protein decreased the incremental PPGR, measured meal GI and meal GL [8], and the fat [9] and viscous soluble fiber [10] were also shown to reduce the glycemic response. The model integrated protein, fat and fiber contents. There was good alignment between the in vivo measurements and the model values; the predicted eGI and eGL and the model can, therefore, be used to predict the GI and GL of processed infant cereal prototypes from the analytical nutrient profiles of the finished product. Knowing the nutrient specification of ingredients and mastering nutrient transformations during processing can also be used to guide product (re)formulation at a very early stage.

### 4.5. Limitations

These data showed that the GI and GL of complete infant cereal prototypes can be predicted from the macronutrient composition of the finished product using the same model that was previously established for breakfast cereals. Although this extends the scope of its usage, the previously discussed limitations of the model remain unchanged: its simplicity captures neither the differences between protein or fat types nor the potential impact of bioactives, such as polyphenols or ferulic acid [33]. Another limitation is that in vivo, the effect of foods on the postprandial insulin response can be assessed, which cannot be estimated using the current model. Although extensive data are available on the GIs of different foods and the potential health impact for adults, much less is known about the applicability of the GI concept in infants/young children. There are known physiological differences between infants and adults, such as pancreatic maturation and function, as well as gastric emptying and transit time [34], which may impact the glucose response, and thus, affect the ability to extrapolate glucose response data from adults to infants. Due to the ethical implications of measuring the PPGR/GI in this age group, at present, our knowledge in this area remains limited and further studies using less invasive measures of PPGR, such as minimally invasive continuous glucose monitoring, are required in infants. Lastly, females were not included in this study. Future studies should include females in order to expand our scientific knowledge of glycemic response in females and to extend the generalizability of our findings.

## 5. Conclusions

By combining data from in vivo studies in healthy adults, we showed that the model previously developed to estimate the GI and GL of breakfast cereals was both accurate and precise for infant cereal prototypes, even though these differed significantly from breakfast cereals in terms of nutrient composition, viscosity, texture and serving size. This model can serve as a simple screening tool to facilitate the development of infant cereal-based products with a PPGR that is more appropriate for health. The model quantifies the impact of not only the glycemic carbohydrates but also of the GI-lowering nutrients, such as proteins, viscous soluble fibers and fats, which represents a significant strength in terms of the applicability of the model for different infant cereal products. Moreover, we provided data to support the association reported in the adult literature between the GL and the measured PPGR.

## Figures and Tables

**Table 1:** Nutritional composition (g/100 g) of 20 samples including 5 precooked, dried cereal flours (A1–A5) and 15 infant cereal prototypes (B1–D5).

	Glucose	Fructose	Maltose	Sucrose	Lactose	Isomaltu.	Starch	CHO	Fib.Sol.	Fib.Ins.	Fat	Protein	Ash	Moisture
A1	0.0	0.0	0.0	2.1	0.0	0.0	49.0	51.1	9.4	6.1	0.6	27.0	3.3	2.5
A2	0.0	0.0	0.0	2.0	0.0	0.0	50.0	52.0	8.9	6.1	0.6	25.6	3.1	3.7
A3	0.0	0.0	3.7	0.5	0.0	0.0	70.9	75.1	4.2	3.6	0.9	10.8	1.3	4.1
A4	0.0	0.0	4.2	0.8	0.0	0.0	70.5	75.5	4.7	2.0	0.8	11.2	1.3	4.6
A5	0.0	0.0	4.1	0.9	0.0	0.0	70.6	75.6	4.5	2.0	0.8	11.2	1.3	4.7
B1	12.7	2.6	6.7	0.0	12.7	0.0	33.5	68.1	3.1	2.0	7.0	15.5	2.1	2.2
B2	1.6	0.0	1.3	10.5	13.1	0.0	40.0	66.5	3.0	2.0	9.4	15.1	2.1	1.8
B3	1.2	3.4	6.6	1.9	6.8	5.1	36.4	61.3	5.4	3.6	14.7	11.1	2.1	1.9
B4	0.7	0.0	7.3	7.9	7.3	4.8	34.1	62.1	5.0	3.4	14.1	11.6	2.1	1.7
B5	0.8	0.0	6.8	10.4	8.5	0.0	37.2	63.6	5.5	3.7	11.1	12.1	2.1	1.9
C1	11.7	0.0	8.9	0.0	13.2	0.0	33.1	66.9	1.7	1.2	8.7	16.3	3.1	2.1
C2	0.0	0.0	1.1	12.1	12.9	0.0	39.2	65.3	2.2	1.5	9.2	14.6	2.9	4.5
C3	0.0	0.0	1.3	11.0	9.2	0.0	39.3	60.8	3.6	2.4	11.5	14.6	2.9	4.3
C4	0.0	0.0	1.2	6.3	9.1	0.0	44.1	60.7	3.5	2.3	12.2	15.3	2.8	3.2
C5	0.0	0.0	1.7	0.7	8.4	0.0	47.5	58.3	4.4	2.9	12.4	16.1	2.8	3.2
D1	0.6	0.0	1.2	10.2	0.6	0.0	44.4	57.0	8.0	4.8	12.6	14.1	2.0	1.5
D2	0.7	0.0	1.4	9.9	7.0	0.0	39.7	58.7	6.0	4.6	12.4	14.0	2.6	1.7
D3	0.7	0.0	1.6	9.2	7.8	0.0	39.5	58.6	6.1	4.2	12.5	14.1	2.6	1.9
D4	0.7	0.0	1.3	9.5	9.6	0.0	38.2	59.3	4.7	3.5	13.8	13.8	2.8	2.1
D5	0.7	0.0	1.5	9.7	12.8	0.0	34.2	59.0	6.1	3.5	12.3	13.8	3.0	2.3

**Table 2:** In vivo estimates (mean ± SE) of the 2h-iAUC, GI and GL and model predictions of eGI and eGL of 20 samples, including 5 precooked, dried cereal flours (A1–A5) and 15 infant cereal prototypes (B1–D5).

	In Vivo Estimates (Mean ± SE)							Model Predictions	
	2h-iAUC (mmol/L × min)	Measured GI			Measured GL (g/50 g)			eGI	eGL (g/50 g)
A1	111 ± 9.5	65 ± 4.9			16.6 ± 1.2			70	17.8
A2	125 ± 10.2	71 ± 5.0			18.6 ± 1.3			71	18.5
A3	171 ± 14.8	91 ± 4.3			34.0 ± 1.6			89	33.3
A4	154 ± 13.3	85 ± 5.4			32.0 ± 2.0			88	33.4
A5	152 ± 10.3	83 ± 4.1			31.2 ± 1.6			89	33.4
B1	177 ± 14.5							72	24.4
B2	148 ± 12.1							67	22.2
B3	130 ± 10.5							64	19.7
B4	132 ± 15.7							65	20.3
B5	151 ± 13.2							69	22.0
C1	210 ± 20.2	73 ± 4.2			24.4 ± 1.4			72	24.2
C2	177 ± 12.5	67 ± 4.9			21.8 ± 1.6			66	21.7
C3	185 ± 17.1	72 ± 5.3			22.0 ± 1.6			66	20.0
C4	176 ± 12.5	69 ± 3.4			21.0 ± 1.0			67	20.5
C5	174 ± 12.0	72 ± 4.3			21.1 ± 1.3			69	20.1
D1	193 ± 18.0	73 ± 5.1			20.9 ± 1.4			69	19.6
D2	177 ± 12.8	68 ± 4.3			19.8 ± 1.3			66	19.4
D3	176 ± 14.1	66 ± 4.7			19.4 ± 1.4			66	19.3
D4	164 ± 12.5	62 ± 4.7			18.4 ± 1.4			65	19.1
D5	167 ± 17.0	62 ± 4.3			18.4 ± 1.3			63	18.4

## Data Availability

Data will be made available upon request to the corresponding author.
